# Differences on Motor Competence in 4-Year-Old Boys and Girls Regarding the Quarter of Birth: Is There a Relative Age Effect?

**DOI:** 10.3390/children8020141

**Published:** 2021-02-13

**Authors:** Rubén Navarro-Patón, Víctor Arufe-Giráldez, Alberto Sanmiguel-Rodríguez, Marcos Mecías-Calvo

**Affiliations:** 1 Facultad de Formación del Profesorado, Universidade de Santiago de Compostela, 27001 Lugo, Spain; ruben.navarro.paton@usc.es; 2 Facultad de Ciencias de la Educación, Universidad de A Coruña, 15008 A Coruña, Spain; 3 Facultad de Lenguas y Educación, Universidad Camilo José Cela, 28692 Madrid, Spain; asrgz2014@gmail.com; 4 Facultad de Lenguas y Educación, Universidad Nebrija, 28015 Madrid, Spain; 5 Facultad de Ciencias de la Salud, Universidad Europea del Atlántico, 39011 Santander, Spain; marcos.mecias@uneatlantico.es; 6 Centro de Investigación y Tecnología Industrial de Cantabria (CITICAN), 39011 Santander, Spain

**Keywords:** relative age effect, schoolchildren, motor competence, manual dexterity, aiming and catching, balance, Movement Assessment Battery for Children-2 (MABC-2)

## Abstract

The aim of this study was to evaluate the differences on motor competence between boys and girls aged 4 years old and investigate the existence of Relative Age Effect on their motor competence. In total, 132 preschool children were evaluated, of whom 60 (45.50%) were girls and 72 (54.5%) were boys. The distribution of the participants was from quarter 1 [*n* = 28 (21.2%)], quarter 2 [*n* = 52 (39.4%)], quarter 3 [*n* = 24 (18.2%)], and quarter 4 [(*n* = 28 (21.2%)], respectively. The Movement Assessment Battery for Children-2 (MABC-2) was used to collect the data. The data show the main effects on quarter of birth factor in manual dexterity (MD; *p* < 0.001), in aiming and catching (A&C; *p* < 0.001), in balance (Bal; *p* < 0.001) and in total test score (TTS; *p* < 0.001). There are also statistical differences on gender factor in MD (*p* < 0.001) and in TTS (*p* = 0.031). A significant effect was also found in the interaction between two factors (gender and quarter of birth) in MD (*p* < 0.001), A&C (*p* < 0.001), and Bal (*p* < 0.001). There are differences in all the variables studied according to the quarter of birth and only in manual dexterity and in the total score if compared according to gender (the scores are higher in girls).

## 1. Introduction

The search for a quality educational system has increased the interest in the evaluation of school performance, which has grown in recent decades [1]. For this reason, tools have been developed to measure it in different countries [2].

The scientific literature indicates that motor competence is the ability of each person to acquire, improve, and execute [3] fine and gross motor skills in an expert way [4], with quality and control of movement [5].

Fundamental movement skills have been classified in the scientific literature as (1) locomotive skills (e.g., running, sliding, jumping); (2) object manipulation and control skills (e.g., hitting, kicking, throwing, and catching); and (3) stability and body control skills (e.g., balance, body rocking) [6].

Motor skills are not an area foreign to the assessment. Thus, recent research shows that the assessment in school physical education is product-focused [7] and moves away from the goal of achieving full development of preschool-age children (that is, physical, cognitive, and social development) [8,9]. It is known that if motor skills are acquired properly during childhood, it will contribute to the acquisition of autonomy, which is understood as the development of the ability to do, be well alone, and live solid relationships with others, in the development of habitual activities (i.e., go to the bathroom, eat alone, move properly, write) [10]. This will help with the later development of more complex and specialized motor skills [6,11,12]. We must bear in mind that many of childhood learning occurs/takes place through motor skills, both fine and gross [13]. Fine motor skills refer to precision movements that involve few muscle groups in the hand, feet, or face. These skills can be performed with or without integrating a visual stimulus. Some activities for assessing fine motor coordination (without integration) are finger movements, threading beads, or inserting coins into a piggy bank [14]. On the other hand, writing or copying shapes and letters are activities that are used for evaluating integration [15]. Gross motor skills require the use of large muscle groups in movements that involve many sections of the body or the entire body. These can be classified into locomotives (running, jumping, sprinting, etc.), manipulative (throwing, receiving, hitting, etc.) and equilibrium (static and dynamic) [6].

The scientific literature has shown that the motor development of boys and girls can be influenced by the environmental context and by sociodemographic factors (i.e., gender or age) [4]. Maturation within the same cohort is another factor to take into account. In areas such as education, the groups are made by chronological age, and therefore, there could be students with up to twelve months of chronological age difference in the same cohort [16]. Therefore, these age differences can generate differences in maturity and experience among the members of a class group [17]. With respect to gender, it is known that boys perform better in general skills compared to girls [18,19,20,21], while girls perform better in fine motor skills [18,21,22], although they have also been reported results in which there were no differences as indicated [23,24]. Studies have also been found in which girls perform better in balance [20,21,22,25], while others report that girls perform better in motor coordination than boys [26,27]. Lastly, a recent systematic review reports the lack of consensus on this [28].

These discrepancies with respect to gender may be due to the fact that in these studies, the relative age of the children has not been taken into account, understanding by Relative Age Effect (RAE) the fact by which children born earlier, in the year calendar within a cohort, perform better than those born later [29]. These differences may also be due to different sports practices in different geographic areas (for example, baseball in the United States) that help or difficult the development of certain types of skills [30]. It may also be due to the fact that physical activity contexts, such as school physical education, have influenced the differences [31]. In Physical Education, the physical maturity that students reach by the mere fact of being born earlier gives them an advantage over those born in the first months of the year [32,33]. In this sense, RAE and the state of biological maturity are closely related, since the former can be an indirect indicator of the potential state of maturity [34]. The school categorizes by chronological age, so the skeletal age of preschoolers may vary due to this categorization [3]. Furthermore, this categorization could influence biological maturation and, consequently, the presence of RAE when motor competence is evaluated [34,35,36]. Recent studies do not show conclusive results on motor competence in relation to age, as some indicate that children born in the first quarter of the year have better motor performance and competence [37,38,39], and others indicate inverse results [40]. These ambiguous results, in ages 3 to 6 years, may be due to individual differences in the development of motor skills in age and sex [9,22,41]. Furthermore, the scientific literature shows that schoolchildren who are relatively younger than their peers are more likely to have/achieve poorer academic results [39,42,43], worse physical condition [33], and less participation in school sports activities [44].

Different standardized tests are used to assess motor competence, including the Movement Assessment Battery for Children, Second Edition (MABC-2) [45], by which differences between boys and girls of the same age can be assessed, and which have been shown to be non-uniform throughout this developmental stage [22].

In our country, RAE studies have been carried out but not in schoolchildren of these ages and, worldwide, to date, few studies have researched the influence of gender and relative age on motor competence in early childhood. Therefore, the objective of this study was to evaluate the differences in motor competence between 4-year-old boys and girls and to investigate the existence of the Relative Age Effect on their motor competence. We address these goals through the following research questions: (1) Are there differences in gender-based tests of motor competence in 4-year-old preschool children? (2) Is RAE present in the global percentile of motor competence in 4-year-old preschool children according to the quarter of birth? Is REA inversely proportional to quarter of birth within the same cohort in the overall total score and in the total percentile? (3) Is RAE present in boys? Is it present in girls?

## 2. Materials and Methods

### 2.1. Study Design

A non-experimental cross-sectional descriptive design was carried out [46]. The variables of the Movement Assessment Battery for Children-2 (MABC-2) were the dependent variables, comparing them according to gender and quarter of birth.

### 2.2. Participants

A non-probabilistic selection of the sample was made, according to the subjects and the geographical proximity of 4 public education centers of Galicia (Spain).

A total of 172 4-year-olds preschool children were invited, of which 15 were excluded for not providing the informed consent signed by their parents or legal guardians, 25 for presenting significant motor skill difficulties (once the battery test began), because the students were below the 5th percentile of the battery. Finally, the sample consisted of 132 preschool children.

All preschool children were classified into quarters based on their trimester of birth [quarter 1 (q1: born from January to March); quarter 2 (q2: born from April to June); quarter 3 (q3: born from July to September) and quarter 4 (q4: born from October to December)] and gender group (boys and girls).

### 2.3. Tools

The Movement Assessment Battery for Children-2 (MABC-2), adapted to the Spanish context by Graupera and Ruíz [47] was used. It has proven to be feasible and reliable to identifying changes in motor skills over time in preschoolers. This battery comprises the following eight standardized tests in three specific skills: manual dexterity (MD) (coins insertion, threading beads, and drawing a trail); aiming and catching (A&C) (catching a bean bag and throwing a bean bag onto mat), and balance (Bal) (one-leg balance, walking with heels raised, and jumping on mats) [45].

This tool provides direct and scalar scores for each test, scalar scores for the dimensions with equivalent percentiles, and a total test score (TTS) with its scalar and percentile equivalent score.

### 2.4. Procedures

The administration of the educational centers was contacted and explained the objective of the study. Once it was done, the teachers of preschool children were included in this explanation. Subsequently, a written document was sent to the parents and/or legal guardians, explaining the objective, purpose, design, and procedure of the study (data recording, analysis techniques and their subsequent use), the declaration of confidentiality, the voluntary participation, and the possibility of withdrawing the child from the study at any time they wish.

Once accepted by the parents and/or legal guardians of the schoolchildren, the necessary sociodemographic data (age, date of birth, sex) were recorded, and the MABC-2 battery was administrated. Each child was evaluated by trained evaluators and individually, wearing comfortable clothing, in the school environment. Standardized equipment was used. The evaluators followed the same methodology in all schools, as well as the instructions in the examiner’s manual for the battery itself. It should be noted that before performing each of the tests of the MABC-2 battery, the students had a practice test, where the examiner can correct possible errors. On the other hand, no instructions were given during the test performing.

After all the tests were completed, the scalar scores for each of the tests, the scalar and percentile scores for all three dimensions (total manual dexterity score, total aiming and catching score, and total balance score), and total test score with its scalar score and equivalent percentile were determined.

All research was carried out in accordance to Declaration of Helsinki. Research protocol was sent to the Ethics Committee of the national EDUCA platform for review and its approval. The protocol was approved with the code number 22019.

### 2.5. Statistical Analysis

For the sociodemographic data analysis, the variables were expressed using frequency tables for categorical variables and central tendency measures for quantitative variables (mean and standard error of mean). The differences in all the variables of the MABC-2 battery across the categories of quarter of birth (q1 vs. q2 vs. q3 vs. q4) and the gender (boys vs. girls) were evaluated using a multivariate analysis of variance (MANOVA). The size of the effect was calculated using partial eta squared (η^2^), and the interaction between variables was calculated using the Bonferroni statistic to learn of the significance. The analysis of variance (ANOVA) was made in order to observe what happened with the total percentile score factor and the quarter of birth in boys, girls, and total sample, through the Welch statistic due to the lack of homoscedasticity and also the post hoc test using the Bonferroni statistic to study the peer significance. SPSS software (SPSS v.25, IBM Corporation, New York, NY, USA) was used for all statistical analyses. The level of significance was set at *p* < 0.05. 

## 3. Results

In total, 132 healthy preschool children were evaluated, of whom 60 (45.50%) were girls and 72 (54.5%) were boys. The distribution of the participants was from quarter 1 [*n* = 28 (21.2%)], quarter 2 [*n* = 52 (39.4%)], quarter 3 [*n* = 24 (18.2%)] and quarter 4 [*n* = 28 (21.2%)], respectively.

**Figure 1:**
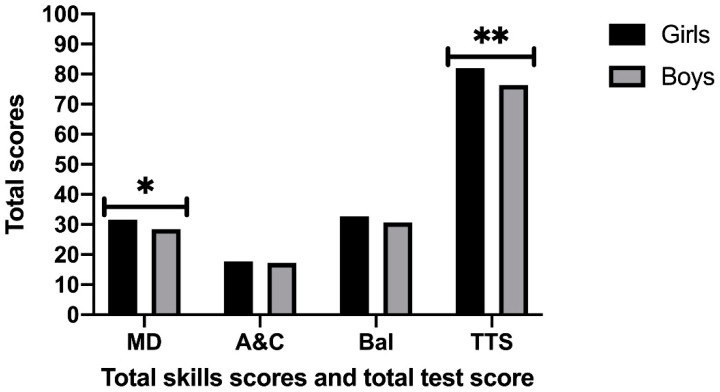
Total Skill Scores and Total Test Score regarding gender. Note: MD: Manual dexterity; A&C: Aiming and Catching; Bal: Balance; TTS: Total Test Score. * Significant differences *p* < 0.001; ** Significant differences *p* < 0.05.

**Figure 2:**
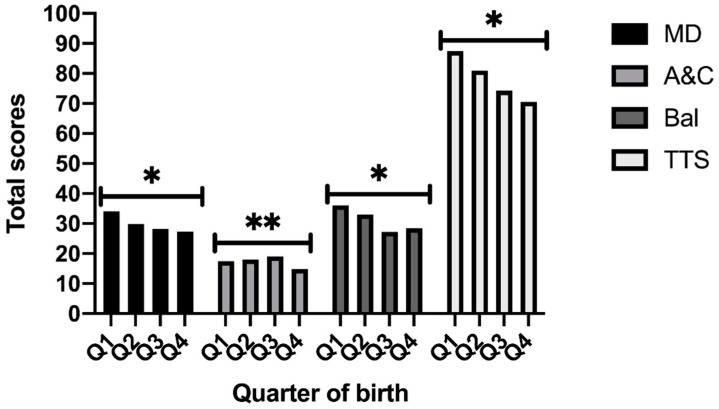
Total Scores regarding skills and quarter of birth. Note: MD: Manual dexterity; A&C: Aiming and catching; Bal: Balance; TTS: Total Test Score; Q: Quarter of birth. * Significant differences *p* < 0.001; ** Significant differences *p* < 0.05.

The results of the MANOVAs (Figure 1 and Figure 2) regarding the manual dexterity (MD) indicated that there is a significant main effect on the quarter of birthdate factor [F (3, 124) = 10.760, *p* < 0.001, η^2^ = 0.21], which is higher in those born in the first quarter, and in the gender factor [F (1, 124) = 14.977, *p* < 0.001, η^2^ = 0.11], being higher in girls than boys. A significant effect was also found in the interaction between both factors [F (3, 124) = 13.490, *p* < 0.001, η^2^ = 0.25]. 

Regarding aiming and catching (A&C), the findings indicated that there is a significant main effect in the quarter of birthdate factor [F (3, 124) = 3.145, *p* = 0.028, η^2^ = 0.07], with higher scores in those born in the second and third quarter but not in gender factor (*p* = 0.068). Statistical differences have been found in the interaction between both factors [F (3, 124) = 7.452, *p* < 0.001, η^2^ = 0.15].

Regarding balance (Bal), the results of the MANOVA indicated that there is a significant main effect of the quarter of birthdate factor [F (3, 124) = 25.840, *p* < 0.001, η^2^ = 0.38], with the scores higher in those born in the first quarter, but not in gender factor (*p* = 0.626). Interaction effects have also been found between both factors [F (3, 124) = 11.703, *p* < 0.001, η^2^ = 0.22]. 

The results with respect to the total test score (TTS) indicated that there is a significant main effect of the quarter of birthdate factor [F (3, 124) = 16.765, *p* < 0.001, η^2^ = 0.29], with higher scores achieved by those born in the first quarter again. A main effect in the gender factor has also been found [F (1, 124) = 4.735, *p* = 0.031, η^2^ = 0.04], being higher scores in girls.

Regarding the comparison by pairs, with respect to the MD (Table 1), statistically significant differences have been found between boys and girls in the first quarter (*p* < 0.001), with higher scores in girls, and in the third (*p* = 0.003) and the fourth quarter (*p* = 0.003), with higher scores in boys. Regarding the A&C, statistically significant differences have been found between boys and girls, being greater in girls in the first (*p* = 0.012) and in the fourth quarter (*p* = 0.002). When Bal is analyzed, statistically significant differences have also been found between boys and girls, in favor of those girls in the third quarter (*p* < 0.001) and in favor of boys in the fourth quarter (*p* < 0.001). Regarding the TTS, the results in the first quarters appear higher scores in girls (*p* = 0.007).

In the pairwise analysis based on gender and the quarter of birth, with respect to the MD in girls, differences were found between q1 vs. q2 (*p* < 0.001), q1 vs. q3 (*p* < 0.001), and q1 vs. q4 (*p* < 0.001). With respect to the boy’s analysis, differences were found between q2 vs. q4 (*p* < 0.001) and q3 vs. q4 (*p* = 0.004). In the A&C, no differences were found in girls. In boys, only significant differences have been found between q1 vs. q2 (*p* = 0.023), q1 vs. q3 (*p* = 0.010), q2 vs. q4 (*p* < 0.001), and q3 vs. q4 (*p* < 0.001). In the Bal for girls, there are differences between q1 vs. q4 (*p* = 0.001), q2 vs. q4 (*p* < 0.001), and q3 vs. q4 (*p* < 0.001). In boys, there are significant differences between q1 vs. q3 (*p* < 0.001), q1 vs. q4 (*p* = 0.002), q2 vs. q3 (*p* < 0.001), and q3 vs. q4 (*p* < 0.001). In the TTS, significant differences were found in girls between q1 vs. q2 (*p* < 0.001), q1 vs. q3 (*p* = 0.029), q1 vs. q4 (*p* < 0.001) and q2 vs. q4 (*p* = 0.023). In boys, there are only significant differences between q2 vs. q3 (*p* = 0.002) and q2 vs. q4 (*p* < 0.001).

**Figure 3:**
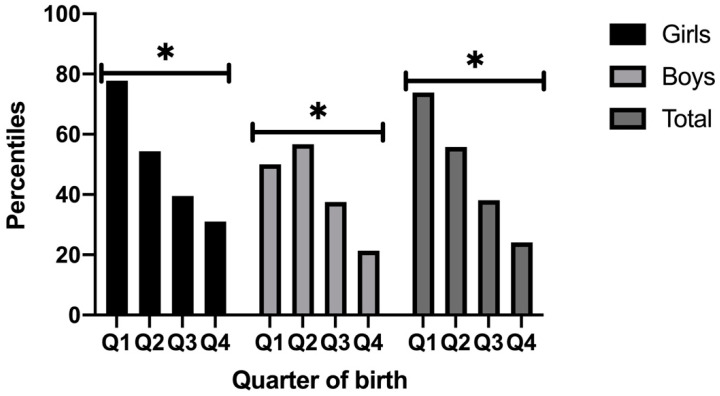
Percentiles according to gender and quarter of birth and global. Note: Q: Quarter of birth; * *p* < 0.001 different between quarter 1 vs. 2 vs. 3 and vs. 4.

Although there are differences between the percentile reached by boys and girls when the quarters are compared, the trend within each gender group indicates that there are statistical differences between students born in q1 (M = 77.83, SE = 2.45) vs. q2 (M = 54.40, SE = 5.71) vs. q3 (M = 39.50, SE = 8.88) vs. q 4 (M = 31.0, SE = 2.26; (*p* < 0.001). The same occurs in boys, q1 (M = 50.00, SE = 0.0) vs. q2 (M = 56.68, SE = 3.57) vs. q3 (M = 37.50, SE = 6.87) vs. q 4 (M = 21.40, SE = 1.01; (*p* < 0.001). The global results show the same trend as if girls and boys are studied independently [i.e., q1 (M = 73.85, SE = 2.81) vs. q2 (M = 55.80, SE = 3.08) vs. q3 (M = 38.16, SE = 5.34) vs. q 4 (M = 24.14, SE = 1.26; (*p* < 0.001)] (Figure 3).

## 4. Discussion

The aim of this study was to evaluate the differences in motor competence between boys and girls aged 4 years old and research the existence of Relative Age Effect in their motor competence.

To answer the first research question, we must indicate that analyzing each of the skills assessed with the MABC-2 battery, with regard to manual dexterity (MD), the results of our study show that girls obtain higher scores than boys, coinciding with the results of previous studies [18,21,22]. This could be related to the type of stereotypical activities that girls carry out, such as writing [48], in addition to being explained by the greater social support and internal motivation in favor of girls participation in hand–eye activities and fine coordination [49]. If we analyze the data obtained in our research on A&C, our results show significant differences between boys and girls born in the first and last quarters of the year [22,24], with better results achieved by girls. This is in contrast to the results of other studies that indicate that boys score higher [18,19,23,25]. No gender differences were found in Bal [22], except when girls and boys are compared in the third trimester (higher in girls) [20,21,22,25], which are results that could be explained by the fact that the girls may have an advantage in terms of developing postural control [25]. The reverse happens when boys and girls are compared in the fourth quarter (highest score in boys) [19]. If we look at the general results of the equilibrium in which no significant differences have been found, it could be argued that the development of this ability does not fully develop until 8–9 years of age [49]. Therefore, we can answer the question posed about in which of the motor coordination tests there are differences, indicating that there are differences in MD and A&C but not in Bal.

In relation to the questions, “Is RAE present in the global percentile of motor competence in 4-year-old preschool children according to the quarter of birth?” and “Is REA inversely proportional to quarter of birth within the same cohort in the overall total score and in the total percentile?”, the data obtained in our research indicate that similar results have been found with respect to gender in terms of the total score and the total percentile. In our study, as in others [22,24], girls born in the first trimester scored higher and are in a higher percentile than boys. The same does not happen in the other quarters of birth, since there are no differences as in other studies [18,19,23,25]. These general gender differences could be due, in part, to the higher scores obtained by girls in the DM and in the Bal [22]. Therefore, for this research questions, we can answer affirmatively.

With respect to the question of whether there is RAE in boys and girls in relation to the quarter of birth, significant differences have been found at the overall and specific level in each of the skills studied using the MABC-2 battery, both in boys and girls, and overall. These scores are higher in boys and girls born in the first quarter compared to those born in the last, by which the RAE is more than evident at these ages in motor coordination tests. In girls, overall scores are higher in those born in the first quarter compared to the second, in those born in the second compared to the third, and in those born in the third compared to the fourth quarter. The same does not exactly happen in boys, since those born in the second quarter are those that obtain the highest scores, followed by those born in the first, those of the third, and those of the fourth. If the data obtained in the entire sample are analyzed, the trend is the same as girls, by which the RAE is evident. These results are similar to those obtained in studies on motor performance [17,33,50,51], selection or detection of sports talents [52,53], where those born in the first months of the year have better motor performance and thus are selected and participate in professional leagues in each of the sports more often [54,55]. Lastly, it should be noted that some studies confirm percentages of motor coordination disorders of 6.56% in 5-year-old boys, with girls obtaining significantly higher motor competence scores in the areas of manual dexterity and balance, while boys score higher, although not significantly, in the area of aiming and catching [56]. For all these reasons, we can answer affirmatively to the question of whether RAE is present in motor competence in 4-year-old preschool children based on the quarter of birth and if it is inversely proportional to the quarter of birth. We can also answer that RAEs exist both in boys and girls, and globally.

Regarding the limitations of this study, we want to point out that the practice of preschool sports and that their body mass indexes (weight and height) have not been taken into account in this research. Furthermore, the sample was not chosen randomly but rather focused on the subjects we had access to, and the sample size was not large and representative enough. As such, the results should be taken with caution to generalize.

From an educational point of view, and specifically in school physical education, although there are significant differences between boys and girls at the global level in the first quarter of birth, these differences do not occur in the rest of the quarters, by which it should be noted that there are no differences between 4-year-old boys and girls in terms of motor skills at the general level. However, it is necessary to reflect on the results that indicate that schoolchildren born earlier in the school year (q1) have an advantage in motor skills compared to those born later (q3 or q4). Therefore, this research invites us to rethink the type of evaluation carried out by physical education teachers, often through standardized tests, which could benefit those born in the first quarters and harm the youngest within the same cohort. In this sense, several strategies are proposed to take into account to implement Physical Education interventions according to our research questions: (1) designing and implementing Physical Education sessions based on the student’s motor competence levels, individualizing learning. Boys and girls achieve motor competence unevenly in different tasks (manual dexterity, grasping and throwing, and balancing) [20,21,22], and younger ones have less motor competence than older ones (within the same cohort) [29,38,39]; (2) design and implement Physical Education curricular tasks with a logical and organized progression that creates a challenge (we must propose tasks that are achievable by all: boys, girls, adults, and children) [57]; (3) increase motivation in physical education classes, contributing to the success of the proposed tasks (tasks should be attractive to everyone, respecting their individual tastes and motivations); (4) the time of motor experiences is a determining factor in the development of motor competence [58], so it is necessary to allow more free time to play and specific physical education during the school day for preschool children (variety of materials and appropriate practice locations) [6,59,60]; (5) use other school environments, such as recess and classroom breaks to carry out Fundamental Movement Skills-based programs [61], since the more time of practice, the better motor competence [32,33].

Future studies should enquire into the RAE effect on other educational stages within the physical education and school sports area.

## Figures and Tables

**Table 1:** Results of Movement Assessment Battery for Children-2 (MABC-2) test based on sex and the quarter of birth.

		Quarter 1 (Born from January to March)		Quarter 2 (Born from April to June)		Quarter 3 (Born from July to September)		Quarter 4 (Born from October to December)	
		Mean	SEM	Mean	SEM	Mean	SEM	Mean	SEM
Total score of manual dexterity	boys	28.00	1.21	29.37	0.43	29.25	0.60	26.40 **^,^***	0.54
	girls	35.00 ^†^	0.49	30.6 *	0.54	26.00 *^,^**^,†^	0.86	29.50 *^,^***^,†^	0.85
	Total	31.50	0.65	29.99	0.34	27.62 *^,^**	0.52	27.95 *^,^**	0.51
Total score of aiming and catching	boys	13.00	1.88	18.87 *	0.66	19.75 *	0.94	13.40 **^,^***	0.84
	girls	18.16 ^†^	0.76	16.80	0.84	17.50	1.33	18.50 ^†^	1.33
	Total	15.58	1.01	17.83	0.53	18.62	0.81	15.95	0.78
Total score of balance	boys	38.00	1.19	33.12	0.67	24.75 *^,^**	0.95	30.20 *^,^***	0.85
	girls	35.66	0.73	32.80	0.85	32.00 ^†^	1.35	24.00 *^,^**^,^***^,†^	1.35
	Total	36.83	1.03	32.96 *	0.54	28.37 *^,^**	0.83	27.10 *^,^**	0.80
Total test score	boys	79.00	3.32	81.37	1.17	73.75 **	1.66	70.00 **	1.48
	girls	88.83 ^†^	1.35	80.20 *	1.48	75.50 *	2.35	72.00 *^,^**	2.35
	Total	83.91	1.79	80.78	0.94	74.62 *^,^**	1.44	71.00 *^,^**	1.39
Total percentile score	boys	50.00	9.5	56.68	3.37	37.50 **	4.75	21.40 *^,^**	4.25
	girls	77.83 ^†^	3.88	54.40 *	4.25	39.50 *	6.72	21.40 *^,^**	6.72
	Total	63.91	5.10	55.70	2.69	38.5 *^,^**	4.09	26.20 *^,^**	3.95

Note. SEM = standard error of mean; * *p* < 0.05 different to quarter 1; ** *p* < 0.05 different to quarter 2; *** *p* < 0.05 different to quarter 3; ^†^ *p* < 0.05 different to boys.

## Data Availability

The data presented in this study are not available in accordance with Regulation (EU) of the European Parliament and of the Council 2016/679 of April 27, 2016 regarding the protection of natural persons with regard to the processing of personal data and the free circulation of these data (RGPD).
